# Non-Bisphosphonate Inhibitors of Isoprenoid Biosynthesis Identified via Computer-Aided Drug Design

**DOI:** 10.1111/j.1747-0285.2011.01164.x

**Published:** 2011-09

**Authors:** Jacob D Durrant, Rong Cao, Alemayehu A Gorfe, Wei Zhu, Jikun Li, Anna Sankovsky, Eric Oldfield, J Andrew McCammon

**Affiliations:** 1 Department of Chemistry & Biochemistry, University of California San Diego9500 Gilman Drive, Mail Code 0365, La Jolla, CA 92093, USA; 2 Department of Chemistry, University of Illinois, Urbana–Champaign600 South Mathews Avenue, Urbana, IL 61801, USA; 3 University of Texas Medical SchoolHouston, 6431 Fannin Street, Houston, TX 77030, USA; 4 Center for Biophysics and Computational Biology, University of IllinoisUrbana–Champaign, 607 South Mathews Avenue, Urbana, IL 61801, USA; 5 Department of Chemistry & Biochemistry, NSF Center for Theoretical Biological Physics, National Biomedical Computation Resource, University of California San DiegoLa Jolla, CA 92093, USA; 6 Department of Pharmacology, University of CaliforniaSan Diego, La Jolla, CA 92093, USA; 7 Howard Hughes Medical Institute, University of CaliforniaSan Diego, La Jolla, CA, USA

**Keywords:** dehydrosqualene synthase, farnesyl diphosphate synthase, isoprenoid biosynthesis, molecular dynamics, presqualene diphosphate, squalene synthase, undecaprenyl diphosphate synthase, virtual screening

## Abstract

The relaxed complex scheme, a virtual-screening methodology that accounts for protein receptor flexibility, was used to identify a low-micromolar, non-bisphosphonate inhibitor of farnesyl diphosphate synthase. Serendipitously, we also found that several predicted farnesyl diphosphate synthase inhibitors were low-micromolar inhibitors of undecaprenyl diphosphate synthase. These results are of interest because farnesyl diphosphate synthase inhibitors are being pursued as both anti-infective and anticancer agents, and undecaprenyl diphosphate synthase inhibitors are antibacterial drug leads.

Over 55 000 naturally occurring isoprenoids have been identified (1). These compounds, the products of the mevalonate, non-mevalonate, and isoprenoid biosynthesis pathways, have diverse functions including visual pigmentation, endocrine signaling, signal transduction, and cell membrane/cell wall biosynthesis (2). Owing to the diversity of isoprenoid products, isoprenoid biosynthesis is the target of several FDA-approved drugs, including treatments for high cholesterol (statins), cancer (taxol), and bone diseases (bisphosphonates) (3). Inhibitors of the isoprenoid biosynthesis pathways are also effective against trypanosomes, including *Trypanosoma cruzi*, the organism responsible for Chagas’ disease, and *Trypanosoma brucei*, the organism responsible for human African sleeping sickness (4–16), as well as against bacteria such as drug-resistant *Staphylococcus aureus*, an ever increasing public-health threat (17).

Two interesting new anti-infective targets involved in isoprenoid biosynthesis are farnesyl diphosphate synthase (FPPS) and undecaprenyl diphosphate synthase (UPPS) (18–21). FPPS catalyzes the condensation of dimethylallyl diphosphate (DMAPP) with isopentenyl diphosphate (IPP) to form geranyl diphosphate (GPP) and thence, farnesyl diphosphate (FPP) (22), while UPPS elongates FPP *via cis* double-bond addition to produce undecaprenyl diphosphate (UPP) (Figure 1) (19). Both enzymes are essential for bacterial cell growth, and UPPS is of particular interest because it is absent in humans.

**Figure 1:**
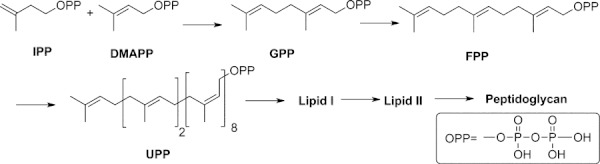
Selected steps in the isoprenoid biosynthetic pathway.

Here, we first describe a virtual-screening approach used to identify novel, non-bisphosphonate FPPS inhibitors, thought to be less vulnerable to rapid removal from the circulatory system *via* bone mineral binding than their bisphosphonate counterparts. Next, we show that these compounds also inhibit two bacterial UPPS enzymes, suggesting a new route to polypharmacophoric, combined FPPS/UPPS inhibition.

## Methods and Materials

### Molecular dynamics (MD) methodology

The initial model for an MD simulation of FPPS was derived from chain A of a *T. brucei* FPPS structure (PDB ID: 2EWG) (22). To calculate the partial charges of the minodronate ligand, Gaussian 03 revision B.04 (Gaussian, Inc.) was used to first minimize the ligand coordinates (6-31G* basis set). A grid potential was then calculated from the resulting structure. The grid potential was subsequently processed with the RESP program (Amber 4.1) for a restrained charge fitting. Antechamber was used to generate additional ligand parameters. To maintain the coordination of the Mg^2+^ with the ligand phosphate groups, as well as to maintain the protein–ligand–Mg^2+^ charge interaction, distances between a number of atom pairs were restrained to the crystallographic values using a force constant of 50 kcal/Å^2^ (Figure S1).

The protein was geometry optimized for 2000 steps *in vacuo* by applying 250 steps of steepest descent, followed by 1750 steps of conjugate gradient, with SANDER (23). The protein active-site Mg^2+^ and ligand were then loaded into Xleap (23) with the ff99SB force field, and the system was solvated and neutralized. The resulting system contained 20 481 water molecules and 13 Na^+^. A two-step minimization (500 steps of steepest descent, followed by 1500 steps of conjugate gradient) was then used to relax the system, first with the protein restrained (force constant 200 kcal/Å^2^) and then with all atoms free. This minimization was followed by 50 ps of NPT simulation with protein restrained (force constant 200 kcal/Å^2^) to equilibrate the solvent, followed by another 100 ps of NPT simulation with the protein free to adjust the system density.

The production run was executed under the NVT ensemble at 300 K. Periodic boundary conditions were used. The cutoff for the non-bonded interactions was 8 Å, and the cutoff for the non-bonded list update was 10 Å. The SHAKE (24) algorithm was used to constrain bonds with hydrogen atoms. A time step of 2 fs was selected. The production simulation ran for 40 ns.

### Clustering

From the last 32 ns of the MD simulation, 1601 frames at regularly spaced intervals were extracted. These frames were aligned by the protein Cα atoms and clustered by root mean square deviation (RMSD) conformational clustering using GROMOS++ (25). The hydrogen bond networks of the members of the three most populated clusters were subsequently inspected to verify that each cluster was structurally distinct. The set of the central members of each cluster constituted an ‘ensemble’ of protein conformations, representative of the many conformations sampled during the MD simulation.

### Virtual-screening protocol

The FPPS crystal-structure used for docking was prepared from 2EWG (22), a structure deposited in the RCSB Protein Data Bank (26). Hydrogen atoms were added to chain A and associated water molecules using the PDB2PQR server (27,28). Other FPPS protein structures were extracted from the MD simulation described earlier. The UPPS structure was obtained from an MD simulation that has been described previously (29).

The receptor structures were processed with the AutoDockTools (ADT) (30) receptor preparation script, which also computed Gasteiger charges. The FPPS partial charges of the active-site Mg^2+^ were ultimately set to +1.5 *e* for docking and to 0.0 *e* for subsequent rescoring. The FPPS and UPPS affinity-map grids were 37.50 Å× 41.25 Å × 37.50 Å and 40.125 Å × 40.125 Å × 40.125 Å, respectively. Both were centered on their respective active sites and had 0.375 Å spacing. For each protein receptor, the appropriate affinity maps were calculated to accommodate the atom types of all library ligands.

Ligands were processed with ADT to add missing hydrogen atoms, to compute Gasteiger partial charges for each atom and to merge non-polar hydrogen atoms. For some compounds, hydrogen atoms were added or removed as needed by Discovery Studio (Accelrys) or Maestro (Schrodinger), followed by a geometry optimization. All torsion angles were assigned with AutoTors (31), enabling full-ligand flexibility.

To identify AutoDock parameters best suited for FPPS, we first selected four known inhibitors: minodronate (**1**), [1-phosphono-2-(pyridin-2-ylamino)ethyl]phosphonic acid (**2**), [2-(dimethyl-lambda∼4∼-sulfanyl)-1-hydroxyethane-1,1-diyl]bis(phosphonic acid) (**3**), and [1-hydroxy-3-(methyl-(4-phenylbutyl)amino)-1-phosphono-propyl]phosphonic acid (**4**) (Table 1). Both the AutoDock parameters as well as the partial charges assigned to the active-site Mg^2+^ were varied systematically, and the known inhibitors were docked into their respective FPPS crystal structures (PDB codes: 2EWG, 2I19, and 2P1C). A parameter set was identified that could recapture the crystallographic poses of **1**, **2**, **3**, and **4**, with RMSD values of 0.81, 1.98, 1.26, and 3.20 Å, respectively. The docking of **4** was the least accurate, perhaps because **4** has many rotatable bonds and interacts with the protein largely through non-specific hydrophobic contacts. Nevertheless, visual inspection revealed that **4** occupied the correct geometric space, and the nitrogen atom and phenyl ring were correctly positioned.

**Table 1:** Positive controls used in the virtual screens (compounds **1–4**) and the compounds tested experimentally against farnesyl diphosphate synthase and undecaprenyl diphosphate synthase (compounds **5–22**)

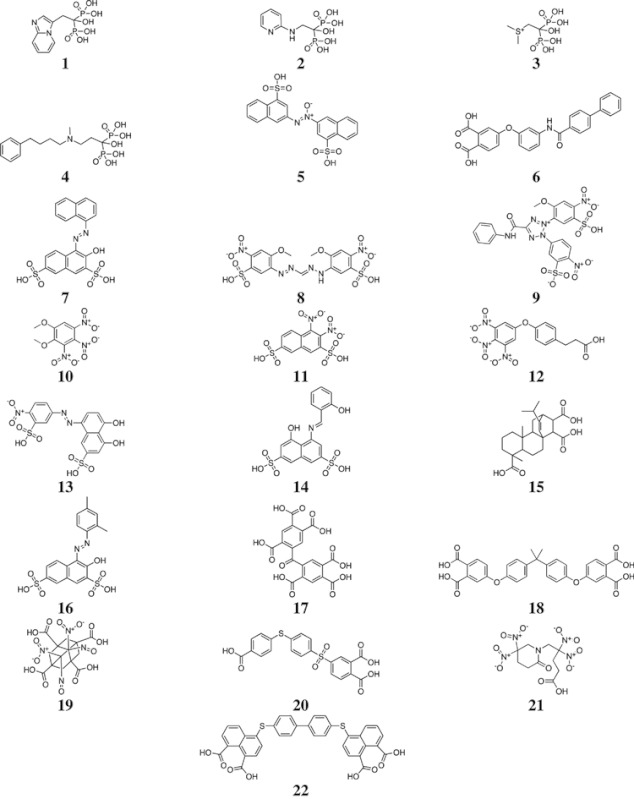

The following docking parameters were ultimately selected: population size of 250, 15 × 10^6^ evaluations, 2.7 × 10^4^ generations, and 100 runs. Clustering of the predicted poses was performed with a cutoff of 0.5 Å RMSD. Default values were used for the remaining docking parameters. For consistency, these same parameters were used in UPPS docking. For FPPS, the best predicted binding pose was judged to be that corresponding to the lowest-energy AutoDock cluster. For UPPS, the lowest-energy pose located in a region of known pharmacological significance was selected.

### FPPS ensemble–based compound scoring

Weighted ensemble-average scores for each compound docked into FPPS were calculated according to equation 1:

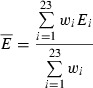


where 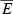 is the weighted ensemble-average score, *w*_*i*_ is the size of cluster *i*, and *E*_*i*_ is the AutoDock score of the compound docked into the centroid of cluster *i*.

### Cloning, expression, and purification of FPPS and UPPS from *S. aureus*

The genes encoding FPPS and UPPS were amplified from *S. aureus* Mu3 genome DNAs using a polymerase chain reaction (PCR). For FPPS, 5′GAC GAC GAC AAG ATG ACG AAT CTA CCG TAC 3′ was used as the forward primer and 5′GAG GAG AAG CCC GGT TAG TGA TCC CTG C 3′ as the reverse primer. For UPPS, the forward primer was 5′GAC GAC GAC AAG ATG TTT AAA AAG CTA ATA AAT AAA AAG AAC AC 3′, and the reverse primer was 5′GAG GAG AAG CCC GGC TAC TCC TCA CTC3′. The amplified FPPS and UPPS genes were purified and ligated into pET-46 Xa/LIC and pET-41 Xa/LIC vectors (Novagen, Madison, WI, USA), respectively. The plasmid with the correct FPPS or UPPS gene was subsequently expressed in *Escherichia coli* BL21 (DE-3) (Novagen). Following cell harvest and lysis, the (His)_6_ tagged FPPS protein was purified by using a HiTrap chelating HP column (GE Healthcare, Piscataway, NJ, USA) and judged by SDS–PAGE to be highly pure. A GSTrap FF column (GE Healthcare) was used to purify the GST-fused UPPS. The fusion tag was then removed by recombinant enterokinase (EMD CROP BIOSCIENCE, INC., Brookfield, WI, USA), and the tag-removed protein was obtained by passing the sample through the same GSTrap FF column. The protein was highly pure according to Coomassie blue–stained SDS–PAGE.

### Assay methodology

The initial screening of FPPS inhibitors obtained from the National Cancer Institute or purchased from Hit2Lead (Chembridge Corporation, San Diego, CA, USA) was carried out using a rapid continuous spectrophotometric assay (32) on 96-well plates with 200 μL reaction mixture (50 mm Tris/HCl, 1 mm MgCl_2_, at pH 7.4, 200 pg FPPS, 200 μm IPP, and 200 μm GPP) in each well. Since there is always the possibility that there could be false positives because of inhibition of the coupling enzymes used in this assay, accurate IC_50_ values were then determined for the initial hits by using a radiometric assay. Briefly, various amounts of potential inhibitor were pre-incubated with FPPS enzymes in a buffer containing 50 mm Hepes, 5 mm MgCl_2_, and 18 μm FPP at pH 7.4. After 15 min, radio-labeled IPP was added, and the reaction was allowed to proceed for 20 min at 37 °C. Reactions were quenched by the addition of 150 μL of HCl/MeOH and incubated at 37 °C for 20 min to hydrolyze the allylic diphosphates. The reaction mixtures were neutralized by the addition of 75 μL of 6 n NaOH and then extracted with 500 μL of hexane. Two hundred microliters of the organic phase was transferred to a scintillation vial for counting. The IC_50_ and *K*_*i*_ values were obtained by fitting the data to a standard rectangular hyperbolic dose–response function in Origin 6.1^a^). To prevent aggregate-based inhibition, 2 mg/mL BSA was included in the reaction mixture, and all reactions were carried out in duplicate.

Activity in UPPS inhibition was again initially screened using a rapid spectrophotometric assay (33). Accurate IC_50_ values for the hits were then determined by radiometric assay, as described previously (33). Briefly, these assays were carried out in duplicate in a buffer containing 50 mm Tris/HCl, 1 mm MgCl_2_, 2.5 μm FPP, and 25 μm radio-labeled IPP at pH 7.4. After 30 min incubation at 25 °C, the reaction was terminated by adding 200 μL of 0.5 m EDTA, and the reaction product was extracted with 500 μL butanol. 300 μL of the organic (upper) layer was then mixed with 3 mL scintillation cocktail and counted for 1 min in a scintillation counter. IC_50_ values were obtained by fitting the inhibition data to a standard rectangular hyperbolic dose–response function in Origin 6.1^a^. Binding to *E. coli*, UPPS was retested in the presence of 0.1% Triton-X-100, 0.1 mg/mL BSA, and 2 mg/mL BSA to rule out aggregation-based inhibition.

The identities of compounds **5**–**8** (Table 1) were confirmed by mass spectrometry, supporting the conclusion that these compounds are in fact responsible for enzyme inhibition.

## Results and Discussion

The current study describes a virtual-screening approach used to identify novel, non-bisphosphonate FPPS inhibitors. The identified compounds also inhibit two bacterial UPPS enzymes, suggesting a new route to polypharmacophoric, combined FPPS/UPPS inhibition.

### Molecular dynamics (MD) simulations

As the molecular motions of protein receptors play a critical role in ligand binding, we first studied FPPS dynamics before attempting to computationally identify non-bisphosphonate FPPS inhibitors. When a ligand approaches its receptor, it does not encounter a single static structure, but rather an ensemble of structures, both open and closed. Upon ligand binding, the closed conformations are stabilized, and the population of configurations shifts to accommodate the ligand. Additionally, ligand binding may induce new protein conformations, not sampled in the ligand-free (*apo*) state, that facilitate improved protein–ligand interactions (34).

**Figure 2:**
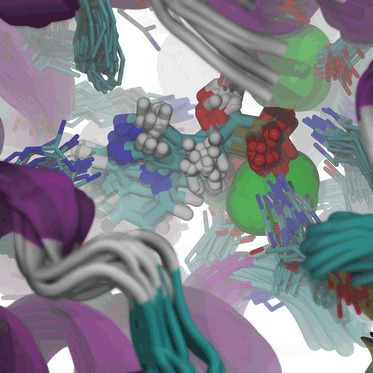
Twenty-three representative protein conformations extracted from an molecular dynamics simulation of farnesyl diphosphate synthase suggest significant active-site flexibility. The protein is shown in ribbon, the ligand is shown in thick licorice, selected active-site residues are shown in thin licorice, and the active-site Mg^2+^ cations are shown as green spheres.

To study protein flexibility, we performed a 40-ns FPPS MD simulation. The protein conformations sampled during the last 32 ns were subsequently clustered into 23 groups by RMSD conformational clustering. A set of 23 protein conformations (an ‘ensemble’), composed of the centroid members of each cluster, was taken to be representative of all conformations sampled. Figure 2 shows these 23 clusters superimposed, demonstrating that the FPPS active site is highly flexible.

### Virtual screening

Past experience has demonstrated that in the case of flexible proteins, accounting for molecular motions is important for predicting ligand binding (35–38). To build upon the protein-flexibility information obtained from the MD simulations, we used the computer program AutoDock 4 (31) to dock the ∼2000 compounds in the National Cancer Institute (NCI) Diversity Set I into various FPPS conformations. Since it was computationally intractable to dock all of these compounds into all 23 protein conformations of the MD ensemble, we instead performed four screens by docking into a representative FPPS crystal structure (*T. brucei* FPPS; PDB ID: 2EWG; (22)) and the top three ensemble conformations, representing 73.1% of the 32-ns MD trajectory. The top 20 ligands from each of the four virtual screens were then compiled into a single list of 73 unique, predicted FPPS inhibitors.

AutoDock 4 (31) was used for ligand docking because it combines a well-tested, physics-based scoring function with a Lamarckian genetic algorithm to improve accuracy (31,39). Although less accurate than thermodynamic integration (31,40), single-step perturbation (41), and free energy perturbation (42), AutoDock provides more rapid estimates of binding energies and performs favorably when compared with other docking programs (43) such as DOCK (44), FleX (45), and GOLD (46).

To further account for protein flexibility, we next used the relaxed complex scheme (RCS) (47), a computational method that combines MD simulations and computer docking, to rerank the 73 candidate inhibitors identified in the preliminary screens. These compounds were docked into the 23 protein conformations extracted from the FPPS MD simulation and then ranked by their ensemble-average docking scores, rather than the docking score associated with a single (e.g., X-ray) structure alone. While the RCS is more computationally intensive than traditional virtual screens, compound ranking is potentially improved. The RCS approach has been used previously to successfully identify several enzyme inhibitors, including inhibitors of FKBP (35), HIV integrase (36), *T. brucei* RNA editing ligase 1 (37), and *Tb*GalE (38).

To expand the list of potential inhibitors, we next considered additional compounds similar to the top 20 of the 73 NCI compounds identified *via* RCS docking. Searches of online databases identified 228 similar compounds from the Hit2Lead database (Chembridge) and 207 additional compounds from the NCI. These compounds were docked as described earlier, again into the top three protein conformations from the MD ensemble and the representative FPPS crystal structure. The top 40 compounds from each of these four screens were then compiled into a single list of 71 unique compounds that were reranked using the RCS described above. Additionally, four positive controls (compounds **1**–**4**, Table 1) were included in the RCS screen. Thus, a total of 148 compounds were scored with the RCS (73 from the NCI Diversity Set I, 71 from the library of similar compounds, and four positive controls).

The FPPS active site contains three Mg^2+^, and AutoDock is known to overestimate binding energies when docking negatively charged ligands into active sites with metal cations (48). In the current study, the active-site Mg^2+^ were initially assigned partial charges of +1.5 *e* because this charge was required to recapture the crystallographic poses of the four positive controls (minodronate, **1**, and **2**–**4**, Table 1). Despite the inaccuracies in the predicted binding energies that likely result from using this partial charge, all four positive controls still ranked in the top 25% of the 148 candidate FPPS inhibitors.

Since overestimating the electrostatic interaction energies might mask the energies of other interactions, we next set the partial charges of the active-site Mg^2+^ to 0.0 *e* and rescored all 148 compounds with the AutoDock scoring function, without redocking. The ensemble-average (RCS) score of the best compound after rescoring was reasonable (−10.9 kcal/mol); however, the positive controls no longer ranked well. To strike a balance, we reranked the 148 compounds again using the average of the scores obtained when the active-site Mg^2+^ had charges of 0.0 *e* and +1.5 *e*. The top 10 compounds from each of these three rerankings were compiled into a single list of 18 unique predicted lead compounds (compounds **5**–**22**, Table 1).

### Experimental screening of FPPS and UPPS inhibition

**Figure 3:**
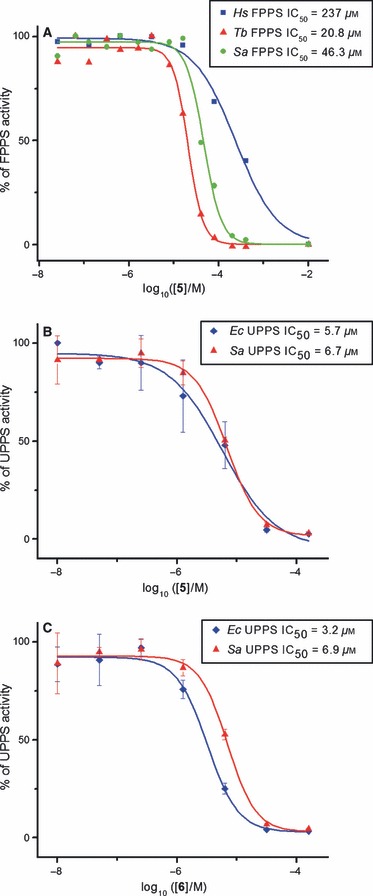
Inhibition of farnesyl diphosphate synthase (FPPS) and undecaprenyl diphosphate synthase (UPPS). (A) Human, *Trypanosoma brucei*, and *Staphylococcus aureus* FPPS inhibition by 5. (B) *Escherichia coli* and *S. aureus* UPPS inhibition by 5. (C) As (B) but inhibition by 6.

We first tested compounds **5**–**22** for inhibition of *T. brucei* (20) and *S. aureus* FPPS. As a counter screen, we also screened for human FPPS inhibition (Table 2). Representative dose–response curves are shown in Figure 3. FPPS inhibition was seen with **5**, (Z)-1,2-bis(4-sulfonaphthalene-2-yl)diazene oxide (*T. brucei* FPPS IC_50_ = 20.8 μm, *K*_i_ = 10 μm; human FPPS IC_50_ = 237 μm, *K*_i_ = 22 μm).

**Table 2:** Farnesyl diphosphate synthase (FPPS) and undecaprenyl diphosphate synthase (UPPS) inhibition by 5–8

	HsFPPS	TbFPPS	SaFPPS	EcUPPS	SaUPPS
	IC_50_ (μm)	IC_50_ (μm)	IC_50_ (μm)	IC_50_ (μm)	IC_50_ (μm)
5	237	20.8	46.3	5.7	6.7
6	>300	>300	>300	3.2	6.9
7	>300	>300	>300	37	16
8	>300	>300	>300	42	12

**Figure 4:**
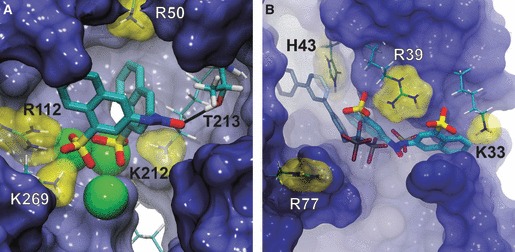
(A) Compound 5 docked into the farnesyl diphosphate synthase ensemble conformation that gave the best AutoDock score when the active-site Mg^2+^ cations were assigned partial charges of +1.5 *e*. Some protein residues have been removed to facilitate visualization. (B) The predicted binding pose of compound 5 similarly docked into undecaprenyl diphosphate synthase.

The best predicted pose of **5** bound to FPPS + Mg^2+^ is shown in Figure 4A. Two sulfonate groups are predicted to interact with the three active-site Mg^2+^, similar to the pose adopted by the two phosphonate groups of known bisphosphonate FPPS inhibitors (22,49–54). Additionally, the oxygen atom of the diazene oxide linker is predicted to form a hydrogen bond with the T213 side chain hydroxyl group. This hydrogen bond is likely strong, because the formal charge on the diazene oxide oxygen atom is −1. Finally, there may be π–cation interactions between one of the naphthalene groups of compound **5** and the positively charged K212 side chain.

As the computational docking results indicated that **5** bound to the allylic binding site of FPPS, it seemed possible that it might also bind to the allylic site of the C_55_ prenyl synthase UPPS, which utilizes FPP as a substrate. To investigate this possibility, **5**–**22** were tested for inhibition of UPPS from *S. aureus* and *E. coli* (Table 2). Several of the predicted FPPS inhibitors did in fact inhibit UPPS. The most potent compound, **6**, had an IC_50_ value of 3.2 μm (*K*_i_ = 0.4 μm) against *S. aureus* UPPS, but no measurable activity (IC_50_ > 300 μm) against human FPPS (Table 2).

A UPPS conformation extracted from a recent MD simulation (29) was then used to better understand the binding of **5**. Compound **5** was docked into the protein conformation with the largest pocket volume because that conformation has been shown to yield the best correlations between activity and docking score (29). The highest-scoring docked pose that placed the ligand within a region of known pharmacological significance is shown in Figure 4B. The two sulfonate groups of compound **5** are predicted to intercalate between three positively charged residues, H43, R39, and K33. Although distant in this particular conformation, perhaps because the original MD simulation did not include a bound ligand, R77 may also participate in ligand binding. Additionally, one of the sulfonate groups of compound **5** is predicted to form a hydrogen bond with N228 (not shown in Figure 4B, for clarity).

A crystallographic bisphosphonate ligand (BPH-608, PDB ID: 2E99) (33) and sulfate ion (PDB ID: 1F75) (2) are also shown in Figure 4B to provide further insight into the predicted binding mode of compound **5**. We note that one of the negatively charged sulfonate groups of compound **5** is near the location where the phosphate groups of known bisphosphonate inhibitors are positioned. Additionally, one of the naphthalene moieties of compound **5** extends in the same direction as the hydrophobic, aromatic rings characteristic of recently identified bisphosphonate inhibitors (33). A crystallographic sulfate ion is positioned near the predicted location of the diazene oxide moiety of compound **5**. Both have electronegative oxygen atoms at the same location, suggesting that this region of the UPPS active site may be well suited to this atom type.

## Conclusions and Future Directions

Compounds **5** through **8** represent interesting leads; all have between 20 and 70 atoms (55) and possess no chiral centers, and all but compound **8** satisfy Lipinski’s rule of five (56). The polar/charged moieties so common in these compounds, thought to be critical for metal binding, may appear at first glance to be uncharacteristic of actual drugs, but the predicted LogP values suggest that only compound **8** has a partition coefficient that is atypical of drug-like molecules (55). The hydrophobic aromatic rings of these compounds may counterbalance any hydrophilic effect, permitting cell membrane permeation. Additionally, there is some precedence for doubly charged inhibitors with intracellular targets; for example, methotrexate, a common cancer drug, contains two carboxylate groups, and a recently discovered trypanocidal compound is doubly sulfonated (57). The chief class of known FPPS inhibitors, bisphosphonates, are also doubly charged.

Compound **5**, active against both FPPS and UPPS, is particularly interesting. It is remarkable that one compound could inhibit two proteins in the same pathway, especially given that FPPS and UPPS share little sequence or structural homology. These two proteins have only 5% sequence similarity according to ClustalW (58) and are not structurally similar according to the FATCAT algorithm (p = 0.0620) (59). The CATH classification (60) of UPPS and FPPS likewise suggests that they are not closely related; UPPS belongs to the ‘alpha beta’ class and has a 3-layer(aba) sandwich architecture, while FPPS belongs to the ‘mainly alpha’ class and has an orthogonal bundle architecture.

Although compound **5** is promising, further lead optimization is clearly needed because the activity of **5** falls far short of the most potent commercially available FPPS inhibitor, zoledronate. Azoxybenzenes and azobenzenes are not particularly drug like, except arguably as prodrugs (e.g., Prontosil), but the diazene oxide linker might be replaced with a sulfonate bridge or, potentially, a ketone or ester linking group.

On the other hand, compound **6** shows promising low μm inhibition of UPPS, but poor FPPS inhibition. This is, of course, of interest from the perspective of anti-infective development, because selective activity against bacterial UPPS combined with poor human FPPS inhibition may result in low human toxicity. Compound **6** is not as potent as tetramic and tetronic acid inhibitors of *Streptococcus pneumoniae* UPPS (IC_50_ ∼ 60–120 nm) (19), although as there has been no optimization of **6**, a more appropriate comparison may be the mean of the IC_50_ values reported previously, ∼7 μm (19). Indeed, the first tetramic acid identified as a UPPS inhibitor (from an experimental high throughput screening study) had an IC_50_ value of 19 μm, so the 3.2 μm IC_50_ value for **6** against *E. coli* UPPS identified from our *in silico* HTS represents a promising lead.

The discovery of low μm, non-bisphosphonate UPPS and FPPS inhibitors is clearly of interest because non-bisphosphonate inhibitors are less vulnerable to rapid removal from the circulatory system by binding to bone mineral, as noted by Jahnke *et al.* (61). Additionally, dual-activity FPPS/GGPPS inhibitors with synergistic activity that allows for a polypharmacophoric, multiprenyl-synthase approach to isoprenoid biosynthesis inhibition have already been designed (52). The current work suggests that a similar approach may be possible for FPPS/UPPS inhibition as well.

## Footnotes

a OriginLab Corp., Northampton, MA, USA, available at: http://www.OriginLab.com

## Abbreviations

DMAPP: dimethylallyl diphosphate
FPP: farnesyl diphosphate
FPPS: farnesyl diphosphate synthase
GPP: geranyl diphosphate
IPP: isopentenyl diphosphate
MD: molecular dynamics
RCS: relaxed complex scheme
RMSD: root mean square deviation
UPP: undecaprenyl diphosphate
UPPS: undecaprenyl diphosphate synthase

## Supporting Information

Figure S1 The restraints used in the moleculardynamics simulation.
