# Supplementary material for: Non-Bisphosphonate Inhibitors of Isoprenoid Biosynthesis Identified via Computer-Aided Drug Design
Source: Chem Biol Drug Des. 2011 Sep;78(3):323–32. doi: 10.1111/j.1747-0285.2011.01164.x (PMC3155669; doi:10.1111/j.1747-0285.2011.01164.x)
Supplement: Figure S1 — The restraints used in the moleculardynamics simulation. [file cbdd0078-0323-SD1.doc]

**
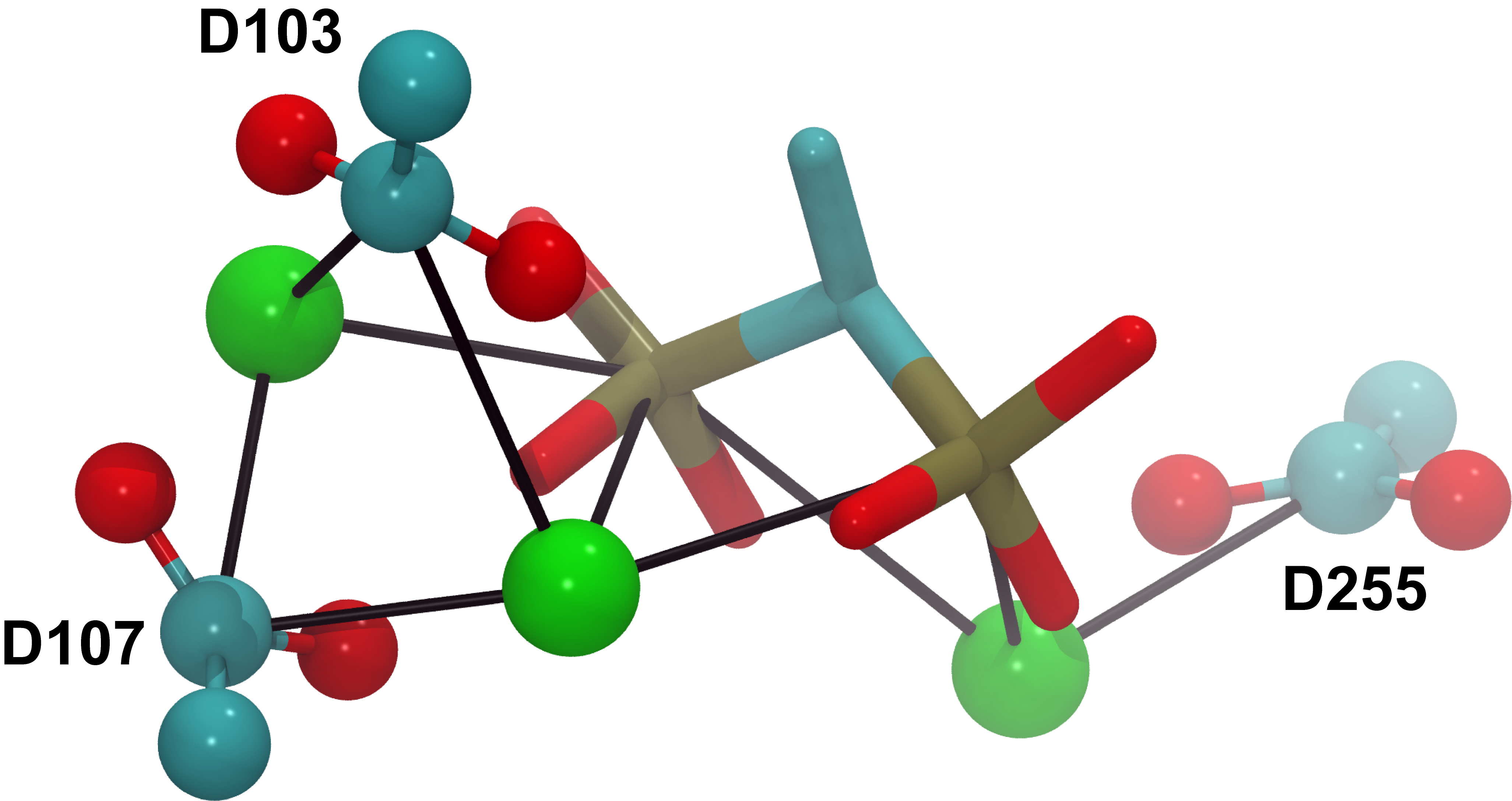
**

**Figure S1**. The restraints used in the molecular dynamics simulation. The side chains of Mg^2+^-coordinating aspartate residues are shown in CPK. A portion of the minodronate ligand is shown in licorice. The three Mg^2+^ cations of the active site are shown as green spheres. Restraints, represented by black lines, were used to prevent the relevant atomic distances from deviating from the crystal-structure values by applying a harmonic potential with a force constant of 50 kcal/Å^2^.
